# Triglyceride-glucose-uric acid (TyG-UA) index as a novel parameter associated with metabolic syndrome among adults: A cross-sectional study

**DOI:** 10.7555/JBR.39.20250347

**Published:** 2026-09-28

**Authors:** Liong Boy Kurniawan, Uleng Bahrun, Mansyur Arif, Agussalim Bukhari, Andi Makbul Aman, Haerani Rasyid

**Affiliations:** 1 Department of Clinical Pathology, Faculty of Medicine, University of Hasanuddin, Makassar, South Sulawesi 90245, Indonesia; 2 Department of Clinical Nutrition, Faculty of Medicine, University of Hasanuddin, Makassar, South Sulawesi 90245, Indonesia; 3 Department of Internal Medicine, Faculty of Medicine, University of Hasanuddin, Makassar, South Sulawesi 90245, Indonesia

Dear Editor,

Metabolic syndrome (MS) is a condition that can cause cardiometabolic disorders, with its incidence increasing in recent decades. It is estimated that a quarter to a third of people worldwide have MS, particularly in developed countries^[1]^. Obesity, insulin resistance, hypertension, and dyslipidemia are common components of metabolic syndrome^[2]^. Several reports have shown a close relationship between hyperuricemia and MS in adolescents, adults, and the elderly^[3–5]^. Elevated levels of blood uric acid are linked to oxidative stress, endothelial dysfunction, hypertension, micro-inflammatory processes, and impaired insulin action, all of which can lead to atherosclerosis, diabetes, insulin resistance, hepatic steatosis, and MS^[6–9]^. High triglyceride levels are also associated with inflammatory conditions, including hypertension, cardiovascular disease, and diabetes^[10–11]^. Several research reports have shown the superiority of the triglyceride-glucose (TyG) index over the homeostasis model assessment of insulin resistance (HOMA-IR) in assessing the risk of MS^[12–13]^. Uric acid is a routinely examined laboratory biomarker and has been associated with MS. However, uric acid is still rarely used in various formulas for identifying MS. Therefore, we propose a novel parameter that incorporates serum uric acid levels into the TyG index formula, called the triglyceride-glucose-uric acid (TyG-UA) index, for identifying individuals with MS.

The present cross-sectional study was conducted from December 2024 to January 2025 at Hasanuddin University Hospital, Makassar, Indonesia. One hundred seventy-four adult volunteers older than 18 years without a history of diabetes mellitus (DM) were recruited. Participants with inflammatory or infectious conditions, as well as those on medications that affect lipids and uric acid, were excluded. Informed consent was obtained from all participants before their involvement. This study was approved by the Institutional Review Board of the Faculty of Medicine, Hasanuddin University (Approval Nos. 1097/UN4.6.4.5.31/PP36/2024 and UH24110978).

Fasting blood samples were collected from the participants' veins, followed by serum separation. ABX Pentra 400 (HORIBA ABX SAS, Montpellier, Occitanie, France) was used to measure glucose, uric acid, triglycerides, and lipid profiles, while insulin was assessed using a Cobas e411 (Roche Diagnostics GmbH, Mannheim, Baden-Württemberg, Germany). HOMA-IR and TyG index were calculated as previously described^[12]^. The TyG-UA index was calculated using the formula: ln[glucose (mg/dL) × triglycerides (mg/dL) × uric acid (mg/dL)/3]. The denominator of 3 was introduced to account for the incorporation of three biochemical components into the composite index. The International Diabetes Federation (IDF) criteria were employed to ascertain MS status as previously described^[1]^.

Continuous variables were assessed for normality using the Kolmogorov–Smirnov test. The chi-square test was used to assess the differences in categorical data. The independent-samples *t*-test (for normally distributed data) or the Mann–Whitney *U* test (for non-normally distributed data) was used to examine the differences in numerical data. The area under the curve (AUC) from the receiver operating characteristic (ROC) analysis was employed to evaluate the ability of the HOMA-IR, TyG index, and TyG-UA index to identify MS. The optimal cut-off value was determined by maximizing the Youden index (sensitivity + specificity − 1). The statistical analysis was carried out using version 21.0 of the Statistical Package for the Social Sciences (SPSS) software.

The characteristics of both MS and non-MS participants are shown in ***Table 1***. The TyG-UA index showed significant correlations with body mass index (BMI), waist circumference (WC), systolic blood pressure, diastolic blood pressure, low-density lipoprotein (LDL), high-density lipoprotein (HDL), triglyceride, fasting plasma glucose (FPG), insulin, and HOMA-IR, with correlation coefficients (*r*) of 0.324, 0.497, 0.360, 0.161, 0.486, −0.449, 0.754, 0.288, 0.364, and 0.406, respectively (all *P* < 0.05). Because triglycerides and glucose are components of the TyG-UA index, their correlations with the composite index should be interpreted cautiously because of mathematical coupling. The results of the ROC curve analysis for determining the ability of HOMA-IR, TyG index, and TyG-UA index to identify MS are shown in ***Fig. 1***. The TyG-UA index (AUC = 0.842, 95% confidence interval [CI], 0.766–0.918, cut-off = 9.8163, sensitivity = 75%, specificity = 83.10%) had a numerically higher AUC than the HOMA-IR (AUC = 0.739, 95% CI, 0.709–0.875, cut-off = 2.6504, sensitivity = 68.80%, specificity = 76.10%) and TyG index (AUC = 0.792, 95% CI 0.646–0.832, cut-off = 8.3717, sensitivity = 75%, specificity = 70.40%) in identifying MS.

**Table 1:** Basic profile of participants' characteristics

Variables	All (*n* = 174)	Non-MS (*n* = 142)	MS (*n* = 32)	*P*
Sex, male [*n* (%)]	86 (49.50)	66 (46.50)	20 (62.50)	0.102^a^
Age (years)	30.50 (20.00–40.00)	30.00 (20.00–39.00)	31.50 (22.00–40.00)	0.545^b^
Body weight (kg)	69.91 ± 15.05	66.64 ± 12.67	84.45 ± 16.33	< 0.001^c^
Height (m)	1.62 ± 0.08	1.61 ± 0.08	1.65 ± 0.09	0.037^c^
BMI (kg/m^2^)	25.51 (16.98–47.61)	24.30 (16.98–40.43)	29.94 (22.64–47.61)	< 0.001^b^
WC (cm)	85.93 ± 11.91	83.10 ± 10.45	98.50 ± 9.76	< 0.001^c^
SBP (mmHg)	120 (80–150)	120 (80–150)	130 (110–150)	< 0.001^b^
DBP (mmHg)	80 (50–100)	80 (50–100)	80 (70–100)	0.001^b^
Total cholesterol (mg/dL)	192.98 ± 36.51	188.58 ± 33.58	218.50 ± 39.46	< 0.001^c^
LDL (mg/dL)	115.60 (46.30–185.00)	111.47 (46.30–181.00)	143.50 (65.00–185.00)	< 0.001^b^
HDL (mg/dL)	44.00 (21.83–76.00)	45.00 (21.83–76.00)	37.00 (26.00–49.30)	< 0.001^b^
Triglyceride (mg/dL)	108.44 ± 38.33	99.97 ± 31.99	146.03 ± 41.90	< 0.001^c^
FPG (mg/dL)	80.75 (56.50–119.80)	80.75 (56.50–112.80)	80.35 (60.00–119.80)	0.575^b^
Insulin (mIU/L)	10.88 (2.59–62.77)	9.87 (2.59–62.77)	16.40 (6.13–35.59)	< 0.001^b^
UA (mg/dL)	5.07 (2.04–10.86)	4.96 (2.04–10.86)	6.16 (3.56–9.70)	< 0.001^b^
HOMA-IR (unit)	2.09 (0.39–16.77)	1.85 (0.39–16.77)	3.48 (0.91–8.84)	< 0.001^b^
TyG index (unit)	8.28 ± 0.39	8.20 ± 0.35	8.62 ± 0.38	< 0.001^c^
TyG-UA index (unit)	9.48 ± 0.56	9.36 ± 0.50	10.06 ± 0.47	< 0.001^c^
^a^Categorical variables are expressed as frequencies (percentages) and compared using the chi-square test. ^b^Non-normally distributed variables are presented as median (minimum–maximum) and compared using the Mann–Whitney *U* test. ^c^Normally distributed variables are presented as mean ± standard deviation and compared using the independent-samples *t*-test. Abbreviations: BMI, body mass index; DBP, diastolic blood pressure; FPG, fasting plasma glucose; HDL, high-density lipoprotein; HOMA-IR, homeostasis model assessment of insulin resistance; LDL, low-density lipoprotein; MS, metabolic syndrome; SBP, systolic blood pressure; TyG index, triglyceride-glucose index; TyG-UA index, triglyceride-glucose-uric acid index; UA, uric acid; WC, waist circumference.				

**Figure 1:**
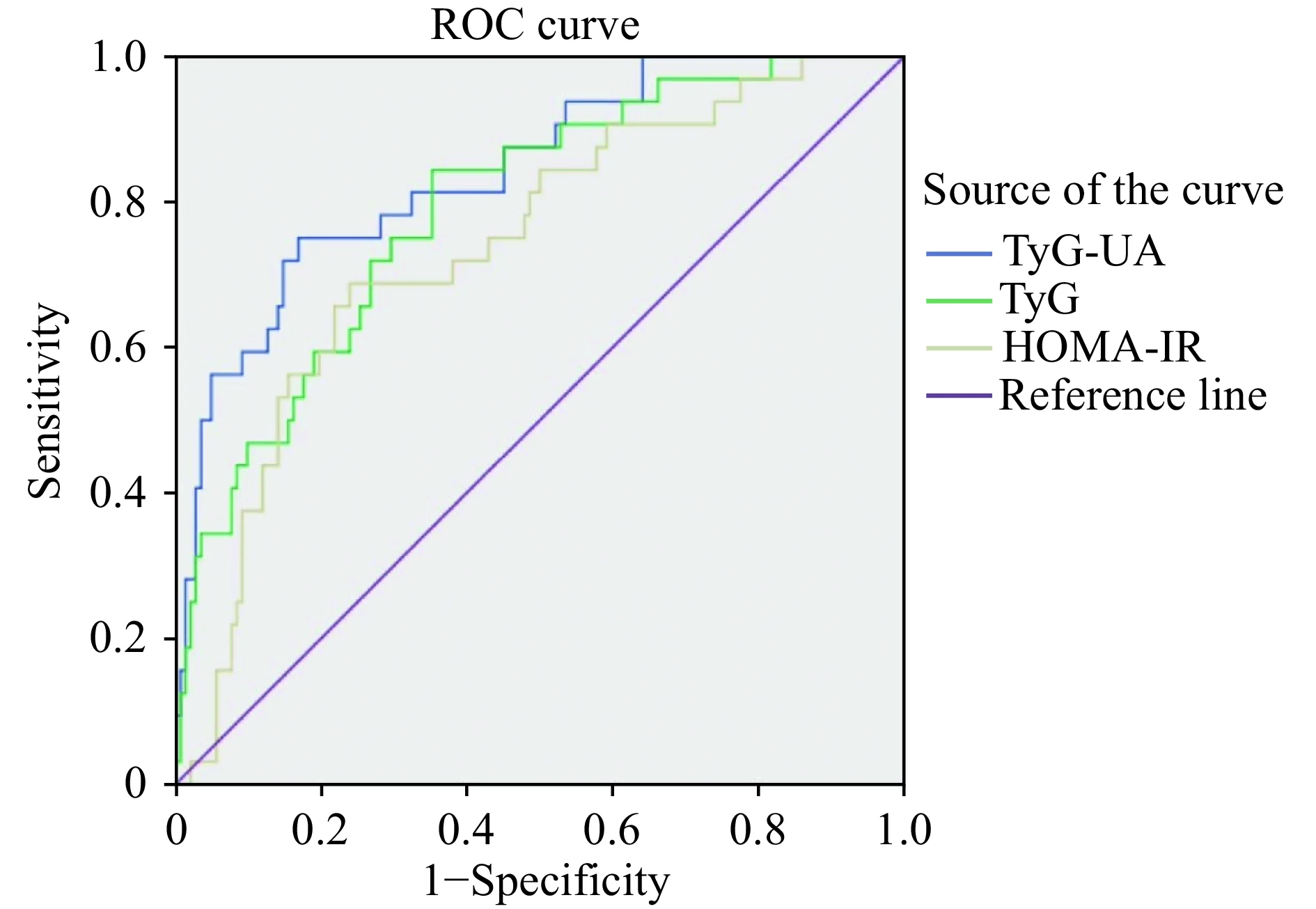
Receiver operating characteristic (ROC) curves of triglyceride-glucose-uric acid (TyG-UA) index and others as determinants of metabolic syndrome (MS). The TyG-UA index shows a numerically higher area under the curve than the homeostasis model assessment of insulin resistance (HOMA-IR) and the triglyceride-glucose (TyG) index.

In the present investigation, the TyG index was modified by incorporating uric acid to create a new index, termed the TyG-UA index. One of the reasons for including uric acid in the index is the presence of various studies and reviews that demonstrate correlations between high levels of plasma uric acid and various cardiometabolic disorders, including MS^[14–16]^. According to Maloberti *et al*^[14]^, MS risk increased by 1.6 times for each 1 mg/dL elevation in blood uric acid level. A study conducted in a population aged > 50 years in Taiwan, China reported a 2.48-fold higher MS risk in participants with high plasma uric acid levels than in those with low levels^[5]^.

In the current study, we demonstrated that the TyG-UA index showed a numerically higher AUC than the TyG index and HOMA-IR in identifying MS. This finding suggests that incorporating uric acid into the TyG index enhances the index's ability to identify the condition of MS. The TyG-UA index combines three parameters: triglycerides, blood glucose, and uric acid, all of which are associated with pro-inflammatory conditions. This modification was intended to improve the discriminatory ability of the TyG index, which only combines two pro-inflammatory parameters. The pathophysiological explanation of uric acid's role (concerning MS components) in identifying MS is described below.

Adipose tissue is one of the tissues proven to play an active role in producing uric acid. Research in mice shows that adipose tissue expresses xanthine oxidoreductase, essential for converting purines into uric acid, thereby enhancing uric acid production in obese mice^[17]^. Uric acid can cause oxidative stress in pancreatic islet cells, leading to impaired insulin secretory function, and in hepatocyte cells, leading to hepatic insulin resistance^[18]^. Oxidative stress due to uric acid can also cause reactive oxygen species (ROS) formation, which can interfere with insulin signaling in target organs, resulting in insulin resistance^[19]^. Increased uric acid levels cause a decrease in nitric oxide (NO) and trigger renin-angiotensin system (RAS) activity, which leads to vasoconstriction. These processes will cause blood vessel remodeling and an increase in blood pressure^[20]^. Experiments using hepatocyte cell cultures showed that uric acid can trigger hepatocyte lipogenesis and lead to increased triglyceride production^[21]^.

To the best of our knowledge, this is the first instance where uric acid parameters have been combined with triglycerides and glucose into one index termed the TyG-UA index. This index showed a higher AUC than the HOMA-IR and TyG index in identifying MS. As a cross-sectional study, it has the inherent inability to elucidate the causal relationships between variables. Since this was a single-center study, future research in diverse populations is required to confirm these findings. Potential confounding by demographic factors, particularly sex, could not be fully excluded and should be examined in larger studies. In conclusion, the novel TyG-UA index showed promising discriminatory ability for identifying MS and had a numerically higher AUC than HOMA-IR and the TyG index in this study. Larger, multicenter prospective studies are needed to validate its performance and determine whether it provides incremental value over established indices.

Yours sincerely,

Liong Boy Kurniawan^1,✉^, Uleng Bahrun^1^, Mansyur Arif^1^, Agussalim Bukhari^2^, Andi Makbul Aman^3^, Haerani Rasyid^3^

^1^Department of Clinical Pathology, Faculty of Medicine, University of Hasanuddin,

Makassar, South Sulawesi 90245,

Indonesia; 

^2^Department of Clinical Nutrition, Faculty of Medicine, University of Hasanuddin,

Makassar, South Sulawesi 90245,

 Indonesia; 

^3^Department of Internal Medicine, Faculty of Medicine, University of Hasanuddin,

Makassar, South Sulawesi 90245,

Indonesia.

^✉^Corresponding author: Liong Boy Kurniawan. E-mail: liongboykurniawan@yahoo.com, ORCID: 0000-0002-2633-0441.
